# From self-assessment to frustration, a small step toward autonomy in robotic navigation

**DOI:** 10.3389/fnbot.2013.00016

**Published:** 2013-10-08

**Authors:** Adrien Jauffret, Nicolas Cuperlier, Philippe Tarroux, Philippe Gaussier

**Affiliations:** ^1^Neurocybertic Team, Equipes Traitement de l'Information et Systèmes Laboratory, UMR 8051Cergy, France; ^2^Cognition Perception et Usages Team, Laboratoire d'Informatique pour la Mécanique et les Sciences de l'Ingénieur Laboratory, CNRS UPR 3251Orsay, France

**Keywords:** bio-inspired robotics, self-assessment, action selection, metalearning, sensory-motor system, neural-networks

## Abstract

Autonomy and self-improvement capabilities are still challenging in the fields of robotics and machine learning. Allowing a robot to autonomously navigate in wide and unknown environments not only requires a repertoire of robust strategies to cope with miscellaneous situations, but also needs mechanisms of self-assessment for guiding learning and for monitoring strategies. Monitoring strategies requires feedbacks on the behavior's quality, from a given fitness system in order to take correct decisions. In this work, we focus on how a second-order controller can be used to (1) manage behaviors according to the situation and (2) seek for human interactions to improve skills. Following an incremental and constructivist approach, we present a generic neural architecture, based on an on-line novelty detection algorithm that may be able to self-evaluate any sensory-motor strategies. This architecture learns contingencies between sensations and actions, giving the expected sensation from the previous perception. Prediction error, coming from surprising events, provides a measure of the quality of the underlying sensory-motor contingencies. We show how a simple second-order controller (emotional system) based on the prediction progress allows the system to regulate its behavior to solve complex navigation tasks and also succeeds in asking for help if it detects dead-lock situations. We propose that this model could be a key structure toward self-assessment and autonomy. We made several experiments that can account for such properties for two different strategies (road following and place cells based navigation) in different situations.

## 1. Introduction

Autonomy, in the field of robotics, is still an open and poorly defined problem for which concepts remain to be invented. By autonomous, we mean a system able to develop and evaluate their skills and decide whether its behavior is relevant or not according to the context. When we talk about autonomy relative to the behavior, we mean the ability to learn behaviors in an open-ended manner but also to manage them. The concepts of open-ended development and cumulative learning have been studied for years in psychology, machine learning and robotics. Those capacities highly depend on intrinsic motivations, involved in exploration and curiosity. The study of intrinsic motivations is gaining more and more attention lately as artificial systems face autonomous cumulative learning problems. Several computational models have been proposed to overcome these problems where some are based on the knowledge of the learning system, while others are based on its competence. The first knowledge-based model, proposed by Schmidhuber (1991), consisted in a world model that learned to predict the next perception given the current one and the action. Prediction progress is used as an intrinsic reward for the system. More recently, a similar model of artificial curiosity proposed by Oudeyer et al. (2007) allows an agent to focus on novel stimuli to improve its learning in challenging situations, avoiding well known and totally unknown ones. The first competence-based model was proposed by Barto et al. (2004) where the intrinsic reward is given on the basis of the inability of the system to reach its goal. In the same way, Baldassarre and Mirolli (Schembri et al., 2007; Santucci et al., 2012) proposed a reinforcement learning architecture that implements skills on the basis of experts. See (Mirolli and Baldassarre, 2013) for a global state of the art of both approaches.

Here, we do not address the problem of learning new skills in a fully autonomous and open-ended manner. We propose in a first step to study open-ended development through the framework of human interaction. In our view, the agent requires a teacher to learn from demonstration but not in a prescriptive way. Since a long time, we develop models allowing robots to learn autonomously different navigation tasks such as: using latent learning to build a cognitive map to be able to reach several goals (Gaussier et al., 2002; Giovannangeli et al., 2006; Laroque et al., 2010; Cuperlier et al., 2007; Hasson et al., 2011; Hirel et al., 2013) or to use explicit or implicit reward to learn different kind of sensori-motor behaviors (Andry et al., 2002, 2004). Yet, it cannot be avoided that at some point the robot fails because of an incomplete learning or because of some changes in the environment. For complex task learning, autonomy means also being able to ask for help and/or to learn from others. It can be made through several ways from stimulus enhancement, response facilitation to different level of imitation. Here we propose to focus on how to allow a system that can be fully autonomous (see previous papers) to work for some time under the supervision of a trainer in order to discover efficiently the solution of a complex problem and/or to complete its learning. To fit natural low level interactions, the training is performed thanks to a leash. Like a dog or a horse, our robot cannot avoid turning its head in the direction of the force applied on the leash. One key difference with a classically supervised system is that the robot has to evaluate when and how to take into account the supervision signal. For sake of simplicity, we will suppose, in this paper, there is no opposition between the robot needs and the trainer requirement. Hence we will not focus on how the robot use latent learning for building some cognitive map or exploit various reinforcement signals to modify its sensory-motor associations [see (Hirel et al., 2013) for the use of a similar architecture focusing on these issues].

Adding self-assessment capabilities to robots should be an interesting solution in this framework of social robotics where humans play a role in the cognitive development of the robot. It could allow the robot to seek for more interactions when needed as in the collaborative control system of Fong et al. (2003). The robot could also communicate its inability to improve its learning. It means that not only does the robot need to code its knowledge but also the limits of its knowledge. This becomes all the more important in integrated robotic systems which have to make decisions based on observations drawn from multiple modalities (Zillich et al., 2011).

Evaluating behavior performance requires the ability to predict the behavior itself at first. Then, it requires to detect potential problems by considering aspects of novelty in these predictions. Novelty is thus an important signal to consider since it represent a key feature providing feedbacks on the behavior's quality. The problem of self-assessment is then sensibly close to the class of novelty detection problems. Novelty detection is a commonly used technique to detect that an input differs in some respect from previous inputs. It is a useful ability for animals to recognize an unexpected perception that could be a potential predator or a possible prey. One of the main goal for self-assessment is self-protection. It is strongly used in situations that caused a failure or a threat in the past, or in the prediction of a threat or a future challenge (Taylor et al., 1995). It reduces the large amount of information received by the animal so that it can focus on unusual stimuli [see (Marsland, 2002) for a global state of the art].

A variety of novelty filters has been proposed where most of them work by learning a representation of a training set (containing only normal data), then trying to underline data that differ significantly from this training set. In the literature, one can find different classes of methods such as statistical outlier detection, novelty detection with supervised neural networks, techniques based on self-organizing map and gated dipole methods.

The standard approach to the problem of outlier detection (Sidak et al., 1967; Devroye and Wise, 1980) is to estimate the unknown distribution μ of a set of *n* independent random variables in order to be able to detect that a new input *X* does not belong to the support of μ. In the same way, extreme value theory (Gumbel, 1958) focuses on distributions of data that have abnormal values in the tails of the distribution that generates the data.

The first known adaptive novelty filter is that of Kohonen and Oja (1976). It proposes a pattern matching algorithm where new inputs are compared with the best-matching learned pattern, meaning that non-zero output is only seen for novel stimuli. Self-organizing networks also provide solutions to detect novelty using unsupervised learning (Kaski et al., 1998) and particularly the so-called Adaptive Resonance Theory (ART) (Carpenter and Grossberg) network that uses a fixed vigilance threshold to add new nodes whenever none of the current categories represents the data. In a sense, the process of the ART network is a form of novelty detection depending on a vigilance threshold.

Supervised neural networks methods propose also novelty detection solutions by recognizing inputs that the classifier cannot categorize reliably. Such methods estimate kernel densities to compute novelty detection in the Bayesian formalism (Bishop, 1994; Roberts and Tarassenko, 1994).

Another solution is given by gated dipole fields, first proposed by Grossberg (1972a,b), then used to compare stimuli and model animal's attention to novelty (Levine and Prueitt, 1992).

Neural models of memory can also detect novelty by learning sequences of states that provide a simple mean of representing pathways through the environment (Hasselmo and McClelland, 1999). Dollé (2011) and Caluwaerts et al. (2012) propose models of metacontroller for spatial navigation that select on the fly the best strategy in a given situation. A competition following by a reinforcement learning allows to associate the action that best fits to the situation. Categorizing contexts are then required to recall the learned action.

Some studies propose that the hippocampus structure, besides its implication in spatial navigation, could be involved in novelty detection, since identifying novelty implies storing memories of normal situations and building expectations from these situations (Knight, 1996; Lisman and Otmakhova, 2001). The importance of novelty in emotional processes was suggested by appraisal theory (Lazarus, 1991; Scherer, 1984; Lewis, 2005). Novelty is closely related to surprise (which could be either positive or negative) but is also determinant in assessment processes for several other emotions (Grandjean and Peters, 2011). Emotional processes are particularly important in decision making while they can guide or bias behavior faster than rational processes, or when rational inferences are insufficient (Damasio, 2003). Griffiths proposes a taxonomy of emotions divided in 2 classes: primary emotions managed by the amygdala and cognitive ones that operate in the prefrontal cortex (Griffiths, 1997).

The role of emotions in communication are also important and have been studied in infants-adults interaction (Tronick, 1989). Infants show self-appraisal capability very early (before the age of 2) while trying to perform a task, but they show a few interest for parents approbation and focus on another goal in case of failure. From the age of 2 the children show reactions (crying, hooking on parents) when facing negative assessment (Stipek et al., 1992; Kelley et al., 2000).

Following the concept of bio-inspired robotics and a constructivist approach, we present integrated robotic control architecture resulting from a close feedback loop between experiments on animals and robots. This leads to a better understanding of the mechanisms by which the brain processes spatial information. In the next sections we first present our previous model of visual place cells that allows a robot to exhibit simple and robust navigation behaviors (Gaussier et al., 2002; Banquet et al., 2005). Since this strategy has been successfully tested in small environments, we met issues while trying to navigate in larger and more complex ones (see Section 2.1.1). We propose to add a second strategy based on a simple, efficient and biologically plausible road following algorithm in order to overcome issues we met with the first one (see Section 2.1.2). Then, we propose a generic neural architecture able to evaluate both sensory-motor navigation strategies (see Section 2.2) based on novelty detection techniques. Finally, we show how a second-order controller, based on the computational literature on intrinsic motivations, can monitor novelty tendencies to modulate both strategies depending on their relevance in a given situation (see Section 2.3). We show how such a controller could also communicates the inability for the robot to perform its task, in order to learn from teacher demonstration. We claim that frustration could be a key feature to improve autonomy in an open-ended manner.

## 2. Materials and methods

### 2.1. Two sensory-motor navigation strategies

Here, we assume that a robot is given a repertoire of behaviors by the designer. In the following, we shortly present 2 of these behaviors that are available to the robot and on which evaluation and regulation mechanisms have been tested.

#### 2.1.1. A model of place cells to perform sensory-motor navigation

In previous works, we developed a biologically plausible model of the hippocampus and entorhinal cortex in order to obtain visual place cells (VPCs) (O'Keefe and Nadel, 1978). VPCs are pyramidal neurons exhibiting high firing rates at a particular location in the environment (place field). Our model allowed controlling mobile robots for visual navigation tasks (Gaussier et al., 2002; Banquet et al., 2005).

**Figure 1:**
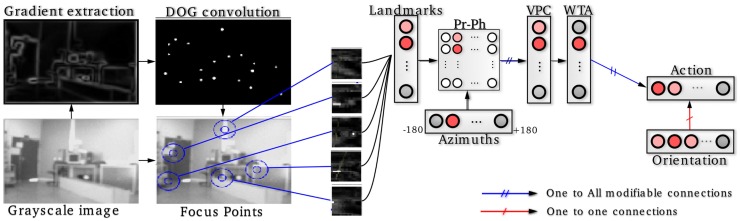
**Sensory-motor model of visual place cells (VPCs)**. Gradient images are convolved with a DoG filter that highlights points of interest on which the system focuses on to extract local views. A VPC learns a specific landmarks-azitmuths pattern. A winner-takes-all competition (WTA) allows to select the winning VPC. Then, an association between the current action (robot's direction) and the winning VPC is learned, after what the system is able to move in such a direction each time that VPC wins.

1. A visual place cell (VPC) learns to recognize a constellation of landmarks-azimuths pattern in the panorama (see Figure 1). VPCs activity depends on the recognition level of corresponding constellations. A winner-takes-all (WTA) competition selects the winning VPC [see (Giovannangeli et al., 2006) for more details].
2. Next, a neural network learns to associate a particular VPC with an action (a direction to follow in our case). The robot perform the action associated with the winning VPC. This sensory-motor architecture [Per-Ac (Gaussier and Zrehen, 1995)] allows the system to learn robust behaviors.

**Figure 2:**
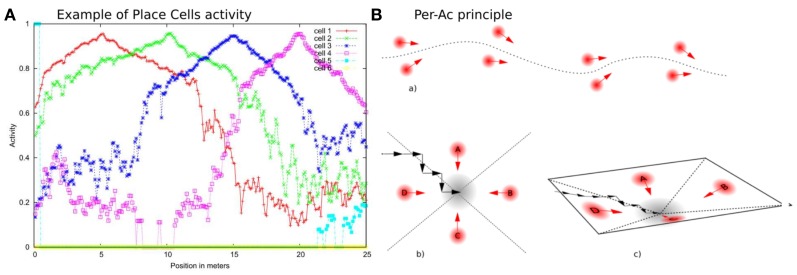
**(A)** Activity of 4 PCs recorded on a linear track in a real outdoor environment. Each maximum of activity corresponds to the learned position of the corresponding PC. Our architecture provides good generalization properties since activities present large place field. **(B)** PerAc principle: Only a few Place/Action associations are needed to perform simple and robust behaviors such as Path learning **(a)** or Homing **(b)**. The agent converges to the goal by falling into an attraction field **(c)**.

VPCs activity, even in outdoor conditions, shows a peak for the learned locations (see Figure 2) and generalizes quite correctly over large distances (2–3 m inside and 20–30 m outside).

Our architecture has been successfully tested in small sized environments (typically one room). However, our visual-only based mechanism shows limitations when trying to scale to larger and more complex environments (multi-room, outdoors). We encounter some situations where the large number of trees all around the system does not leave enough available landmarks to recognize a specific place (the entire panorama is full of green leaves that only represent noise for the system) and the only way to overcome such a problem is to follow the road below. We propose to overcome this issue by adding to our current architecture a biologically plausible road following strategy. Such a strategy allows the robot to follow roads rather than learning Place Cells, in situations where it is neither necessary, nor efficient to do so. Providing two different strategies to the robot is not sufficient by itself to navigate autonomously. The system also needs an action selection mechanism that evaluates both strategies (on the basis of a “meta” learning) to be able to select the right one in a given situation.

#### 2.1.2. A model to perform road following behavior

In previous works, we presented a fast and robust biologically plausible road following strategy (Jauffret et al., 2013). Our algorithm consists in finding the best vanishing point among N potential points in an image. For example, lets consider 5 vanishing points equally distributed on the skyline. The robot will orient itself toward the winning vanishing point.

**Figure 3:**
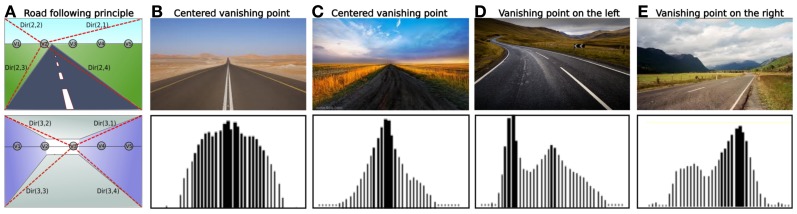
**(A)** Road following principle for 5 vanishing point neurons (from V1 to V5) for outdoor and indoor cases (red dotted line: preferred directions of the winning neuron). Up: V2 is the best candidate. Down: V3 is the best candidate. **(B–E)** Results obtained with our algorithm on real images. For each cases: Up: real image of a road. Down: activity levels of 41 neurons, each one firing for a particular vanishing point location on the image. **(B)** Road with boundaries: the vanishing point is well detected in the center of the image and generalized quite correctly to neighborhood **(C)** Without boundaries: the vanishing point remain salient since there is a significant gradient between road and grass. **(D)** Twisting road: 2 vanishing points are detected, one on the left and one in the middle. Nevertheless, the more active is the one on the left. **(E)** A vanishing point is detected on the right side.

1. The system processes to an edge extraction of incoming images.
2. For each vanishing point considered corresponds one “vanishing” neuron *Vn* that integrates pixels whose edge orientation is aligned to this vanishing point (see Figure 3). The most active vanishing neuron corresponds to that where edges are mostly convergent to.
3. Then, a simple WTA competition selects the best candidate between the N vanishing neurons.

The motor control of our model is directly inspired by control theories of Braitenberg vehicles (Braitenberg, 1986). This control is quite simple: when a vanishing point is detected on the right (resp. left), the robot will turn right (resp. left). Convergent behavior emerges from sensory-motor interactions between the system and its environment, without any need for an internal representation of the environment, or inference. Consequently, angular precision is less important than sample rate in such a control. We tested this algorithm on real images of road (see Figure 3) in several situations.

Our system succeeds in following any types of vanishing points such as roads, corridors, paths or railways. Furthermore, this algorithm has a satisfying framerate of 20 images per seconds (for 41 vanishing point neurons tested on a I7 core processor) and this framerate increases when considering less neurons. Such a high framerate is obtained because only the higher gradients are considered in our algorithm. Therefore, the intensity of the gradient have been normalized by using the cosinus of the angle. So, in Figure 3 (case B) the gradient of road edges is not really high even though the vanishing point is detected.

A drawback of this method is the adjustment of the skyline position. Moreover, in some environments, the vanishing lines above the horizon can be an information (like in a corridor or in forest) although high reliefs or clouds can disturb localization of the vanishing point.

### 2.2. A neural model for novelty detection

**Figure 4:**
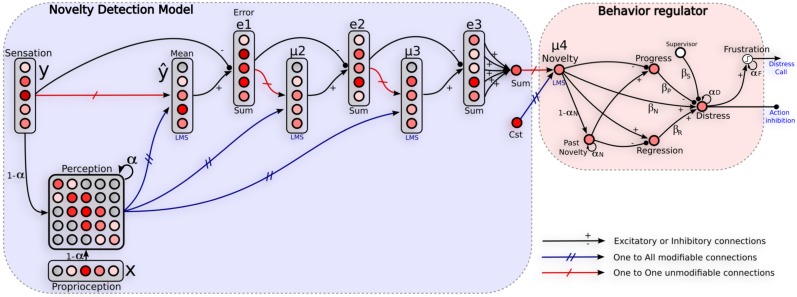
**Neural architecture relying on self-assessment. Left:** network used to detect unexpected events. It learns the sensory-motor contingency of a given strategy by learning to predict the current sensation from the previous perception. Perception is defined as a short-term-memory of recent sensation/action tuples. Such a perception is used to predict an approximation of the error, variance and skewness relative to the sensation *y*. A global novelty level gives to the system a direct feedback on the quality of the behavior involved. **Right:** Emotional controller regulating the behavior. A recurrent neuron (Distress) integrates instant progress and regress of the prediction error, and novelty activity to take into account stagnation of such a prediction error. Actions proposed by the corresponding strategy are inhibited proportionally to the distress level. A neuron (Frustration) fires only when the distress activity reaches a threshold allowing the system to call for help. α_*x*_ and β_*x*_ represent weights of the regulator's parameters.

Here we present a generic model for self-assessment based on novelty detection techniques. Our model consists in two steps. First, the learning of the sensory-motor contingencies induced by the navigation strategy involved in a normal situation (training set), second the ability to detect extraneous sensory-motor patterns in novel situations (see Figure 4).

#### 2.2.1. Modeling the dynamic interaction between the agent and the environment

Learning to predict the sensory-motor contingencies of a strategy can be viewed as finding invariants in the robot's perception. In visual perception (Gibson, 1979), an affordance can be defined as building or accessing to an invariant characterizing one particular sensory-motor behavior. Based from this statement, we consider perception as the result of the learning of sensation/action associations allowing a globally consistent behavior (see (Gaussier et al., 2004; Maillard et al., 2005) for a complete mathematical definition of perception).

Following this assumption, we defined robot's perception as the integral of all its affordances. An affordance referring to a particular sensations/actions state:

(1) $$Per(t)=\int_{-\infty }^{t}Sen^{T}(t).Ac(t).dt$$

Where *Sen* denotes a vector of sensations (sensory input), and *Ac* a vector of actions (given by agent's proprioception).

Lets denote *y* like Sen., a vector of *n* neurons *y*_*i*_ relative to agent's own sensations, *i* ϵ *N*. It can be both place cells or vanishing point cells in our case. *y* can be viewed as a set of random variable *y*_*i*_. *x* is a vector of neurons *x*_*i*_ relative to agent's proprioception, where the winning neuron code for the current orientation. A matrix *Per* estimates the robot's perception by integrating sensations *y* and actions *x* in a finite shifting temporal window defined by the recurrent weight α. *Per* is the tensorial product between *x* and *y* with recurrent connections of weight α. It codes a short term memory of the agent's perception, where *Per*_*i,j*_ denotes the particular tuple of both *x*_*i*_ and *y*_*i*_ neurons:

(2) $$Per(t+1)=\alpha .Per(t)+(1-\alpha ).Sen^{T}(t).Ac(t)$$

(3) $$Per(t)=\sum_{i=0}^{t}\alpha^{i-1}.(1-\alpha ).Sen^{T}(t).Ac(t)$$

Basically, it means that recent inputs have a higher weight in our process than older ones. This type of filter has been tested by Richefeu and Manzanera (2006) in a motion detection context. The parameter α is used in order to attach more importance to the near past than to the far past.

#### 2.2.2. Detecting novelty by processing absolute differences between predicted and real sensation:

Following this internal model of the robot's perception, we defined a vector *ŷ*, same size as *y*, that estimates the mean *E*[*y*] of the current sensation *y* from the perception matrix *Per* by an online least mean square algorithm (LMS) (Widrow and Hoff, 1960): As a classical conditionning (Pavlov, 1927) the vector *ŷ* modifies on the fly the weights of connections coming from the perception matrix (unconditionned stimulus US) in order to estimate the sensation vector *y* (conditionned stimulus CS). We make the assumption that *y* follows a Gaussian distribution required by least-squares. An absolute difference between *y* and *ŷ* defines the instant error vector *e*. In the same manner, a vector ê, estimates the first moment about the mean μ_2_ = *E*[*e*], of the current error *e* = *y* − *ŷ*, from the perception matrix *Per* by an online LMS algorithm. The second-order error is defined as *e*_2_ = *e* − μ_2_. The second moment about the mean is defined as μ_3_ = *E*[|*e* − μ_2_|].

The third order error is defined as *e*_3_ = *e*_2_ − μ_3_. Novelty *N* is defined as the global third moment about the mean. *N* is a single neuron that integrates all *e*_3_ neurons activities: *N* = |∑^*n*^_*i* = 1_ *e*_3_*i*__|. *N* is summarized by:

(4) $$\begin{array}{l} N=E[||(||y-E[y]||-E[||y-E[y]||])-E[||y-E[y]||\\ \;-E[||y-E[y]||]] \end{array}$$

(5) $$where\;||y||=\sqrt{\sum_{i=0}^{n}y_{i}^{2}}(L^{2}-norm)\;or\;\sum_{i=0}^{n}\left|y_{i}^{2}|(L1-norm\right)$$

*N* represents the prediction error of the network, that will be used to detect unexpected events. Here, we defined novelty as the third order moment about the mean for empirical reasons while it is a good trade-off between precision and latency. Here, the different moments μ_2_, μ_3_, and μ_4_ represent respectively the pseudo-variance, the pseudo-skewness and the pseudo-kurtosis while their measure follows the L1-norm rather than the *L*^2^-norm. Our architecture is thus able to learn an internal model of the dynamical interactions the system has with the external world.

### 2.3. Modeling frustration to regulate behaviors and improve learning

We showed that our model for self-assessment is able to give feedbacks on the quality of the behavior of the strategy involved. However, the system was not using such a confidence feedback to regulate its behavior. Here, we propose to implement an emotional controller able to make use of the novelty level, coming from the prediction mechanism. We propose that only considering the absolute novelty level is not sufficient to take correct decisions and regulate behaviors. First, short perturbations (small obstacles, sensor disturbance, visual ambiguities or singular false recognitions) should not affect so much the robot's behavior. Most of the time, the good generalization properties of our sensory-motor strategies allow the robot to stay inside the “attraction field” of the learned behavior (see Figure 2) and thus perform its task correctly, even if unexpected events appear. Because it is more interesting to consider the evolution of such a novelty activity rather than its absolute level, the agent should integrate the novelty activity over time and monitor its evolution to be able to judge its own behavior. If the prediction error remains high or increases whatever the agent tries, then the behavior should be considered as inefficient. And if this inefficiency is lasting this means the agent is caught in a deadlock. Similar assumptions have been proposed by Schmidhuber (1991) in a model-building control system driven by curiosity. This model deals with both problems of (1) do not take into account parts of the environment which are inherently unpredictable and (2) try to solve easy tasks before focusing on harder tasks. The author proposes to learn to predict cumulative error changes rather than simply learning to predict errors.

Based on previous works (Hasson et al., 2011), we propose to compute the instantaneous progress *P*(*t*) = *N*(*t* − δ_*t*_) − *N*(*t*) and the instantaneous regress *R*(*t*) = *N*(*t*) − *N*(*t* − δ_*t*_) as the derivatives of the novelty level *N*(*t*).

Lets define an analog potential of frustration as a recurrent neuron that integrates instant progress and regress (see Figure 4). It also integrates novelty activity *N*(*t*) to take into account stagnation of such a prediction error. This potential of frustration is called the distress level *D*(*t*) in the followings. Actions proposed by the corresponding strategy are inhibited proportionally to this level. Frustration is then defined as a binary decision *F*(*t*):

(6) $$F(t)=\{\begin{array}{l} 1\;if\;D(t)>T\\ 0\;otherwise \end{array}$$

with *T* a threshold parameter, and D(t) the distress level defined as:

(7) $$D(t)=\alpha_{D}D(t-\delta_{t})+\beta_{S}S+\beta_{R}R(t)-\beta_{P}P(t)+\beta_{N}N(t)$$

with S(t) a reward coming from the supervisor and α_*D*_, β_*S*_, β_*P*_, β_*N*_ weights of each variable. The binary frustration neuron fires only when the distress activity reach a threshold *T* (0.9 in our experiments). It allows the system to stop and call for help in order to improve its learning in novel situations.

### 2.4. Selecting and merging strategies with a dynamic neural field

**Figure 5:**
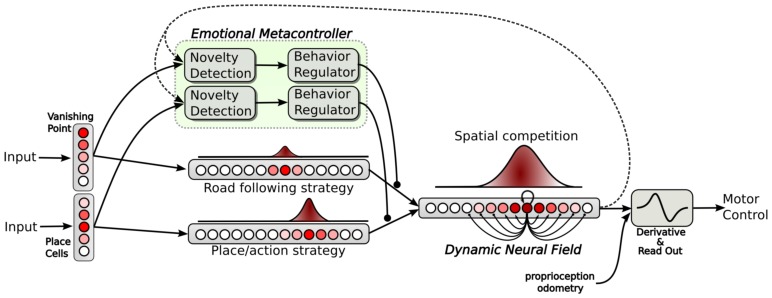
**Neural architecture relying on action selection. Down:** both strategies provide an action in a field (each neuron of the field coding for a particular orientation). All action fields are merged into a dynamic neural field. This neural field provides a solution for decision making by selecting or merging actions in a robust manner (dynamic attractors) and also provides good properties such as temporal filtering. **Up:** an emotional metacontroller learns to predict both strategies from its sensations and from the action proposed by the neural field (feedback link). Distress levels, depending on prediction errors, are used to modulate the action choice.

In our architecture, both strategies (place/action associations and vanishing point following) and their respective metacontroller run in parallel as independent channels (see Figure 5). Each strategy provides an action (an orientation) in a separate field of 361 neurons. Each neuron of the field codes for a particular orientation. Each field of action is inhibited proportionally to the distress level of the corresponding strategy. Both fields are merged into a global Dynamic Neural Field providing solutions for action selection/merging rather than a strict competition (Amari, 1977). The neural fields properties have already been successfully tested to move robot arms by imitation using visual tracking of movement (Andry et al., 2004), or motor control for the navigation of mobile robots (Schoner et al., 1995; Quoy et al., 2003). Neural Fields can account for interesting properties such as action selection according to contextual inputs or persistence in more detailed models (Prescott et al., 2001; Guillot-Gurnett et al., 2002). The neural field equation proposed by Amari is the following:

(8) $$\tau .\frac{u(x,t)}{dt}=-u(x,t)+I(x,t)+h+\int_{z\epsilon V_{x}}w(z).f(x-z,t)dz$$

where *u*(*x*, *t*) refers to the activity of neuron *x* (coding for an angle), at time *t*. *I*(*x*, *t*) is the input to the system. *h* is a negative constant that ensures the stability. τ is the relaxation rate of the system. *w* is the interaction kernel in the neural field activation. A difference of Gaussian (DOG) models these lateral interactions that can be excitatory or inhibitory. *Vx* is the lateral interaction interval defining the neighborhood. Properties of this equation allow the computation of attractors corresponding to fixed points of the dynamics and to local maxima of the neural field activity. Selecting or merging multiple actions depends on the distance between them. Indeed, if two inputs are spatially close, the dynamic gives rise to a single attractor corresponding to the average of them (merging). Otherwise, if we progressively amplify the distance between inputs, a bifurcation point appears for a critical distance. The previous attractor becomes a repeller and two new attractors emerge. Oscillations between multiple actions are avoided by the hysteresis property of this competition/cooperation mechanism. Finally, a simple derivative of the robot orientation allows for motor control of the robot speed [see (Cuperlier et al., 2005) for more details].

### 2.5. Experimental setup

We have tested our models in several situations for both strategies. We first present experiments running in simulation showing the model principles. Next, we present an experiment with a real robot showing how our model deals with known difficulties of real life experiments such as odometry correction, noisy sensors, dynamic of obstacles, people moving, lights changing, etc.

#### 2.5.1. Simulations

**Figure 6:**
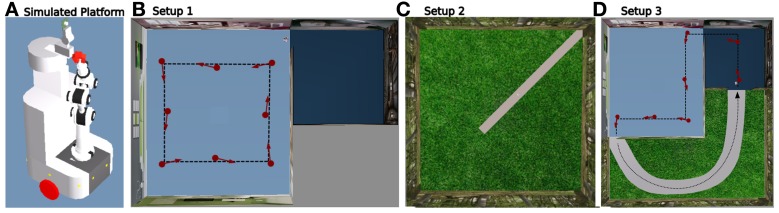
**Setup in Webots simulated environments. (A)** Robotic platform used in our simulations. **(B)** Setup 1 used to test the place/action strategy. The system evolves in a simulated room of 15 x 15 m. It learns a few places associated with different actions (in red) to perform an ideal round behavior (black dotted line). **(C)** Setup 2 used to test the road following strategy. The system evolves in a simulated outside environment of 40 x 40 m and can navigate both on road or grass. **(D)** Setup 3 used to test strategies selection/cooperation. A road links both rooms. No more than 7 place/action associations are needed for the robot to perform the entire loop (black dotted line). It does not need to learn anything off road while following the road is sufficient here.

We used a 40 * 40 cm wide simulated robotic platform (see Figure 6A) equipped with 2 wheels, proximity sensors for obstacle avoidance and a pan-tilt camera used to extract points of interest in the visual panorama and a fixed camera to perceive vanishing points (a copy of our robulab platform from Robosoft). Our simulation software (Webots from Cyberbotics) provides physically realistic model for the robot and obstacles but neither noise nor 3D objects near walls are taken into account (2D realistic snapshots of our lab are simply stuck on simulated walls).

**Setup 1:**

The place/action strategy is put ON while the road following strategy stays OFF. The purpose of the experiment is to test the self-assessment mechanism on the place/action strategy. The environment is a simulated room of 15 * 15 m (see Figure 6B with a uniform floor and salient landmarks on walls. The robot is trained by a human teacher (supervised learning) to perform a round path by learning Place Cell/Action associations. No more than 8 place/action associations were sufficient for the robot to perform a robust round trip in our experiment. A second smaller room is unknown by the system as no places have been learned in it. Consequently, navigating in this room results in inconsistent movements. The evaluation mechanism learns to predict the sensory-motor contingencies of the place/action strategy while the robot performs its round trip in a normal situation (similar to the training set). In this setup, the vector of sensation *Sen* is defined by the vector of 8 Place Cells learned by the system. We set the recurrent weight α = 0.95 empirically, based on the frequency of changes in sensations. The sensory-motor loop of that strategy is quite slow since states only change when the robot navigates from one place to another (it mainly depends on the distance between 2 places and the robot's linear velocity). Indeed, an α near to 1 results in a long temporal window (old states are more important than recent ones). 3 laps were necessary for the evaluation mechanism to completely predict its sensation from all sensory-motor states perceived during the trip. Indeed, learning is completed only when the novelty level reaches a minimum (typically below 0.4) and remains flat in all places.

**Setup 2:** The road following strategy is put ON while the place/action strategy stays OFF. The purpose of the experiment is to test the self-assessment mechanism on the road following strategy. The environment is a simulated garden of 40 * 40 m (see Figure 6C) with a white road passing over grass on the floor and trees texture on walls. The system is able to correctly follow roads when one is in its field of view. On the other side, navigating on grass results in random movements since there is no stable and well-defined vanishing point to follow. The evaluation mechanism learns to predict the sensory-motor contingencies of this strategy while the robot performs road following in a normal situation (training set). In this setup, the vector of sensation *y* is defined by 13 vanishing point neurons processed by the system. We set the recurrent weight α = 0.7 empirically, based on the velocity of the sensory-motor loop. Indeed, the sensory-motor loop of that strategy is significantly faster than for the place/action strategy while vanishing point states change at a speed that directly depends on the robot's angular velocity. 2 min. of navigation were sufficient for the system to completely predict its sensation from sensory-motor situations perceived while following a road. Learning is completed when the novelty level reaches a minimum of 0.4 and stagnates.

**Setup 3:** This time, both strategies are active and run in parallel. The purpose of the experiment is to test strategies cooperation in a complex environment that is a mix of Setup 1 an 2 (see Figure 6D). A road is now linking both rooms by an outdoor part so that the robot can perform the entire loop. The system is trained to perform the loop: passing through both rooms and outside environment. Our model allows the system to correctly perform the entire loop by the learning of only 7 place/action associations. Indeed, the system does not need to learn any place on the outside part while following the road is sufficient in that part to perform the desired task. Consequently, the teacher does not have to correct the system in that part since the behavior resulting from the road following strategy is already the desired one.

#### 2.5.2. Experiment on real robot

**Figure 7:**
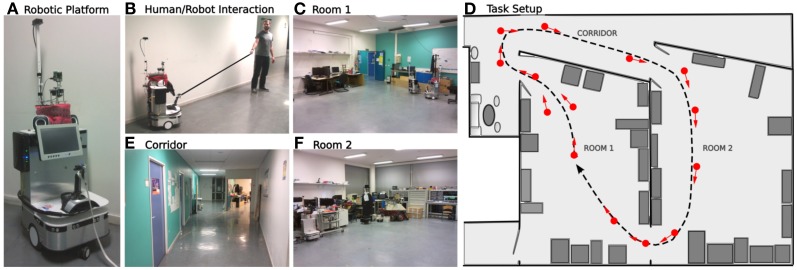
**Experimental setup in our laboratory. (A)** Robotic platform used (Robulab from Robosoft). **(B)** Supervised learning: the teacher uses a leash to pull the robot in the desired direction. The robot learns the path autonomously. **(C–E)** The 3 different rooms of the experiment. **(F)** Learned behavior. The robot learns to travel from room 1, passing through the corridor, to room 2, then back to room 1. About 14 place/action associations (red arrows) are learned to perform an ideal loop (black dotted line).

The following experiment runs in a real indoor environment (part of our laboratory). We used a real robotic platform similar to the simulated one (see Figure 7A). The environment is composed by 2 different rooms and a corridor (see Figures 7C–E). The place/action strategy is put ON while the road following strategy stays OFF. The purpose of the experiment is to test the frustration mechanism on the place/action strategy on a real robot experiment. The task for the robot is to achieve a complete loop passing through both rooms and corridor. 14 place/action were necessary for the robot to learn to perform the loop (see Figure 7F). As a stereotypical human/dog training interaction (Giovannangeli et al., 2006), the teacher uses a leash to pull the robot in the desired direction (see Figure 7B). Thus, the robot is detecting prediction error by comparing human order to its own will. This novelty detection neuromodulates the vigilance of the system so that it decides to recruit a new place cell and learns the association to its current orientation. Following such interactions, the robot is able to learn the path the human is teaching. A proscriptive learning (correcting the system rather than showing it the path) is necessary to get a stable and robust attraction field.

## 3. Results

### 3.1. Results relying on novelty detection experiments

After the system has completely learned the desired trajectory (see Setup 1, Figure 6) and also learned to predict the sensory-motor contingencies relative to this trajectory, we tested it in several situations to show the ability for our model to detect whether such a situation is normal or abnormal.

**Figure 8:**
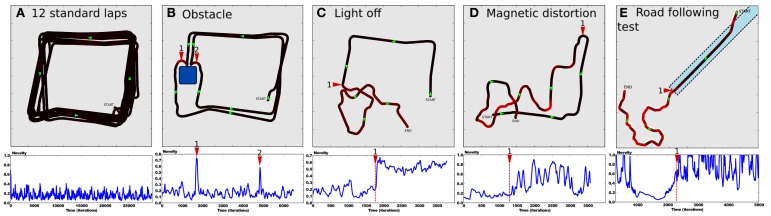
**(A–D)** Results of Setup 1 (see Figure 6). **(A)** Up: trajectory after performing 12 laps autonomously without disturbance. Down:novelty level stays below 0.5 with a constant variance as no abnormal event appears. **(B)** Up: an obstacle suddenly appears in the environment. Obstacle detection allows the robot to avoid it. Generalization capabilities of place cells allow it to go back into the learned path. Down: novelty level shows peaks for respectively the first (1) and second (2) time the system faces the obstacle. **(C)** Up: the system performs its task correctly (from START to 1) when the ambient light is suddenly switched off (1). It results in random movements, as no cues are visible. The robot is totally lost (from 1 to END) Down: novelty mean grows significantly. **(D)** Up: the robot performs its task correctly (from START to 1) when the north direction is suddenly shifted by 90 degrees (1). It results in random movements since unexpected actions are performed in each places (from 1 to END). Down: novelty variance grows significantly. **(E)** Results of Setup 2. Up: the robot starts on grass, converges to the road (dotted line), then follows it until its end. Down: novelty level decreases as the system converges to the road. It grows up progressively at the end of the road (1).

In a first experiment, we tested the robustness of the strategy in a normal situation (see Figure 8A). The robot performs 12 standard laps without disturbance. Results show a robust and stable behavior with a trajectory close to the desired one. The novelty level stays relatively low since it never gets over 0.4, with a mean value of 0.2. It defines the minimum prediction error the system is able to achieve for this task. Such a minimum error is directly linked to the degree of deepness of the prediction process (the *n*th pseudo-moment about the mean). Since we defined the novelty as the third pseudo-moment about the mean, our model is not able to characterize statistical variations over such a precision. A fourth and fifth moment should be able to respectively learn the novelty mean and its variance.

In a second experiment, we introduce an obstacle in the environment so that the robot is forced to avoid it (see Figure 8B). Direct priority is given to the obstacle avoidance strategy by a subsomption architecture. The system avoids the obstacle, then successfully goes back to its original path thanks to the generalization properties of place cells/action associations. Novelty level shows peaks when the robot is avoiding the obstacle, since the orientation taken does not correspond to the learned one in that place.

In a fourth experiment, the light is suddenly put OFF while the robot performs its task (see Figure 8C). Consequently, the visual system is not able to maintain coherent place cells activity and the robot becomes totally lost. It results in random movements. Novelty level shows a sudden offset after the light is put OFF but keeps more or less the same variance. Indeed, the system is not able to recognize places anymore, even if it tries to predict it.

In the same way, a fifth experiment proposes to suddenly shift the north direction by 90 degrees (see Figure 8D). The system performs its task when the north is suddenly shifted. The robot behavior tends to be random a few seconds after the event. The novelty level shows large variations. Indeed, the system sometimes takes an unexpected orientation, sometimes a predicted one.

Finally, a sixth experiment proposes to test generalization capabilities of the novelty detection mechanism on the road following strategy (see Setup 2, Figure 6). The environment contains one road stopping in the middle, and grass elsewhere. The robot starts on grass, in a corner, oriented toward the road (see Figure 8E). Results show that the robot converges to the road in order to be aligned with the road, then it correctly follows it until its end. Finally, it ends its trip by random movements onto grass after leaving the road, as no coherent vanishing point is perceived. Novelty level shows a progressive decrease while the robot converges to the road, then stays minimum and quite stable while following it. Novelty level increases progressively when leaving the road and stays high until the end of the experiment.

We also tested the robustness of the self-evaluation mechanism on a 1 h navigation experiment (not shown here).

### 3.2. Results relying on frustration experiment

**Figure 9:**
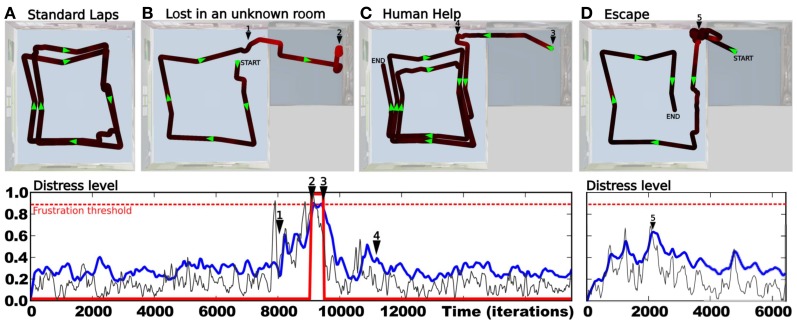
**Simultaion highlighting the need for a frustration mechanism (see Setup 1, Figure 6)**. Up: **(A)** Trajectory after performing some laps autonomously without disturbance. **(B)** The supervisor suddenly interferes to deviate the robot into the second room (1). The robot tries to perform its task without success. The distress level increases progressively while there is no consistency in the perceived sensory-motor sequence. Finally, the system stops and call for help (2). **(C)** The supervisor assists the robot by pulling it in the right direction to escape this room (3). The system learns the new place/action association. Once leaving the small room, it goes back to its stable attractor (4) and perform its task correctly. **(D)** After human demonstration, the simulated robot is able to escape autonomously from the small room. Down:evolution of the distress level in time. It increases as the robot becomes lost in the small room (1). It reaches a frustration threshold of 0.9 (red dotted line) (2), then goes back to normal after human help (4). It increases when the robot is between both rooms (5), indicating that a learning refinement is possible. However stays below the threshold.

In this experiment, we highlight the need for a frustration mechanism to request help in distress situations. The robot has learned to perform a squared loop in a room (see Setup 1, Figure 6). In a first period, the robot performs its task without disturbance (see Figure 9A). Results show a robust behavior with a stable trajectory close to the desired one. The distress level stays relatively low (below 0.4), with a mean below 0.3. It is the minimum prediction error the system is able to achieve for this task in a normal situation.

After some time, the teacher suddenly interferes with the robot to deviate its trajectory toward the second room (see Figure 9B). The robot tries to perform its task by taking the orientation associated with the winning place cell. The distress level increases while there is no consistency in the perceived sensory-motor sequence. After a while, the system stops for a distress call when the distress level reaches a frustration threshold (0.9 in our experiment). The teacher assists the robot by pulling it in the right direction to escape the small room (see Figure 9C). The teacher correction pushes the system to learn a new place/action association and the prediction mechanism to learn to predict this new situation. The robot successfully escapes the small room. The distress level decreases fast because the interaction with the teacher acts as an inhibitory signal in our emotional model. Once leaving the small room, the robot goes back to the first room and converges again to a stable attractor. It continues performing its original task correctly. In another experiment, the robot starts in the small room, in a place different from the learned one (see Figure 9D). Since the robot already faces this situation in the past and thanks to the good generalization properties of place cells, it knows what to do to escape the room and to get back to its stable attractor. Results show that the robot takes the learned orientation to escape the room. The distress level stays low because the situation is considered as normal (predicted) this time. As the robot reaches the frontier between both rooms, its behavior tends to be a bit hesitating. This is due to place ambiguity since the robot hesitates between two place cells associated with contradictory actions. The distress level increases progressively. However, such an odd situation is not long enough to trigger a distress call, and the robot finally successes in getting back to its stable attractor. The distress level decreases slowly and the situation goes back to normal.

Such an interaction allows the system to learn from the teacher how to solve the problem so that it will be able to escape autonomously next time.

### 3.3. Results relying on strategies cooperation experiment

In the following experiments, we highlight the need for an emotional controller to regulate behaviors to solve complex navigation tasks. Navigating in a wide and complex environment requires a metacontroller to make different strategies cooperate in a coherent manner. The robot has learned to perform a complete loop, passing from one room, to the other, to the garden, then back to the first room (see Setup 3, Figure 6). Both strategies and their evaluation mechanisms run in parallel. Strategies cooperate by proposing their actions weighted in real time by their own evaluation. Actions coming from the different strategies are merged into a dynamic neural field that controls robot's movement (see Part.2.4). It allows a smooth cooperation rather than a strict competition. It is also important to notice here that the frustration mechanism does not trigger a distress call procedure in the following experiments. Since we want to test smooth cooperation, the robot does not stop even if both strategies reach the frustration threshold.

**Figure 10:**
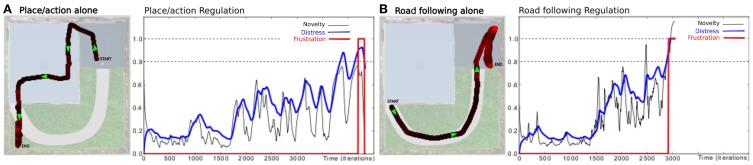
**Unitary tests (Setup 2, Figure 6)**. We tested only one active strategy at a time to be sure that the task can not be solved by a single strategy. **(A)** Trajectory of the robot once the robot has learned to perform the path. It performs its task correctly by the only use of the place/action strategy. Behavior does not stay consistent when entering the outside part while there is no place/action association learned here. The distress level grows and reaches the frustration threshold. **(B)** We bring the robot to the beginning of the road. It follows the road correctly by the only use of the road following strategy. Behavior does not stay consistent when entering the small room while there no consistent vanishing point to follow. The distress level grows and reaches the frustration threshold.

In a first simulation, we tested each strategy alone to ensure the system can not solve such a complex task with only a single strategy. When testing the place/action strategy (see Figure 10A), results show that the robot performs its task correctly in both room, but falls into a deadlock when navigating outdoors and finally get frustrated. In the same way, when testing the road following strategy (see Figure 10B), the robot follows the road correctly in the garden, but falls into a deadlock when entering a room and finally get frustrated.

**Figure 11:**
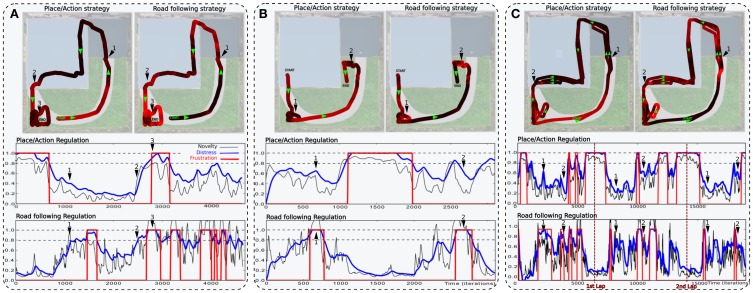
**Simulations highlighting the synergy of cooperating strategies (see Setup 2, Figure 6). (A)** The robot starts on the road and performs one lap autonomously. It fails to finish the loop and falls into a deadlock (3). The distress level shows the cooperation of both strategies. The place/action strategy is strongly inhibited as the system navigates outside (start to 1) and strongly active inside (1–2). Conversely, the road following strategy is strongly active outside (start to 1) but almost inhibited inside (1–2). At the end, the robot leaves the inside part but fails to converge to the road because of its orientation (2–3). **(B)** In another example, the robot successes in following the road after a few hesitation (1), but fails in entering the small room (2). **(C)** The robot performs 3 laps. (1) and (2) indicates respectively each times where the robot get in and outside. Results show that distress levels of both strategies are, most of the time, opposite in phase. Ends of each lap are indicated by vertical red-dotted lines. The behavior is mostly driven by the place/action strategy in both rooms but driven by the road following strategy outdoors.

In a second simulation, the robot performs one lap autonomously (see Figure 11A). It starts in the middle of the road at the bottom of the environment. It performs the loop correctly but fails to finish it and falls into a deadlock when entering the garden. It is due to the orientation the robot takes when leaving the room. If this orientation is too much different from the direction of the road, the system is not able to converge to it.

The distress level shows the cooperation of both strategies during the loop. The place/action strategy is strongly inhibited as the system navigates in the garden and strongly active inside. Conversely, the road following strategy is strongly active when navigating on road outside but almost inhibited inside.

In a third simulation, the robot starts in the first room, successes in converging to the road after a few hesitation, but fails to enter the small room (see Figure 11B). The reason for this success in converging to the road this time is mainly by chance. Next, the robot fails to enter the small room because the generalization properties of place cells allow the robot to recognize this room before entering in it. As a result, it decided to turn too soon and falls into a local minimum. Such a problem can be solved by learning a new place/action association at the end of the road, ensuring to correctly enter the room.

In a fourth experiment, the robot performs 3 laps autonomously (see Figure 11C). The robot starts in the middle of the road at the bottom of the environment. Results show that, most of the time, the distress levels of both strategy are opposite in phase. The distress level of the place/action strategy stays low indoors as the sensory-motor sequence stays predictable. It is high outside while no discriminant landmarks are recognized and no places have been categorized in that part. On the other hand, one can see that the distress level of the vanishing point following strategy is low when the robot follows the road outside. It is mostly high indoors while there is no consistent vanishing point to follow. Distress levels induce proportional inhibition of corresponding behaviors. Accordingly, the robot's behavior is mostly driven by the vanishing point following strategy while navigating on the road outside. Conversely, it is mostly driven by the place/action strategy on the inside part.

Beyond such predictable results, the experiment exhibits good properties that emerge from the synergy of both strategies. As a matter of fact, we encounter several situations where the cooperation enhance the performance obtained with a single strategy. It usually happens in situations where a place/action association allows the robot to pass through a door. In several cases, the contrast induced by an open door make it be perceived as a coherent vanishing point by the system so that the robot naturally converges in its direction without the need for multiple and precise place/action associations. Despite the fact that such a property increases the quality of the behavior, it may be a constraint in others.

Finally, our results also underline some issues during transitions from a place/action to a road following strategy. Indeed, the teacher has to be careful pulling the robot in the direction of the road, otherwise the system can not evaluate the vanishing point correctly and allow the robot to follow the road. It is due to the delay the controller needs to evaluate a strategy. This can results in a deadlock situation where the system switches from one strategy to another without being frustrated enough to call for help.

### 3.4. Results relying on real robot experiment

**Figure 12:**
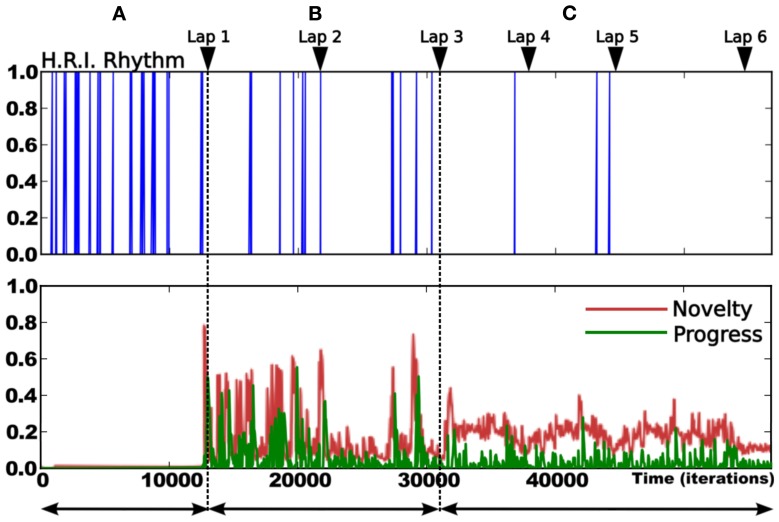
**Details of the learning stage over 6 laps**. Up: rhythm of human/robot interactions (HRI): Dirac pulses correspond to guiding instants. The frequency of interaction decreases over time. It gives a direct measure of the system's autonomy (inversely proportional to frequency). Down:novelty (red) and Progress (green) level. We observe 3 different periods: **(A)** corresponds to the beginning of the learning session (first lap). The high frequency of pulses indicates that the human teacher is roughly directive as the robot does not know anything about the task. The metacontroller is OFF during this stage. **(B)** corresponds to the evaluation stage (second and third lap). The teacher evaluates the robot's behavior and correct it only when needed. The metacontroller is ON and starts to learn to predict the sensory-motor sequences. Consequently, both novelty and progress levels are high during this stage. **(C)** corresponds to the final stage where the robot is autonomous enough to stop learning. Rare corrections are still needed at some points.

The following experiments were performed in our laboratory using a robot similar to the simulated one (see Figure 7). The purpose of the experiment is to test the frustration mechanism on the place/action strategy on a real robot experiment (person and furniture moving, ambient light changing). The road following strategy is disabled. The robot has learned to perform a complete loop, navigating from the first room, to the corridor, to the second room, then back to the first one. The prediction mechanism starts to learn to predict the place/action contingencies after the system finishes the first lap (see Figure 12). We choose not to let the prediction mechanism learn during the first lap in order to get a stable behavior before the system tries to predict it.

**Figure 13:**
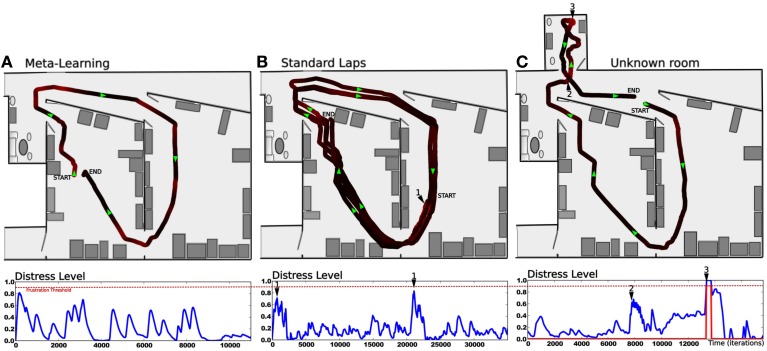
**Results relying on real robot experiment (see Figure 7). (A)** 1 lap trajectory during the metalearning stage. The robot has already learned to perform the task and learns to predict it. The distress level is high each time the robot get from one predicted state to a unknown one. Then the distress level goes down as the robot learns to predict that unknown state. All sensory-motor states are almost predictable after one lap. **(B)** After the metalearning stage, we let the robot perform some laps autonomously. The distress level stays low except in one particular place (1) which corresponds to a place where the robot is close to the window and is slightly disturbed by the sunset. The robot is less confident in this area, however it is not sufficient to frustrate it. **(C)** Later, the robot performs its task when the supervisor suddenly interferes to deviate the robot into an unknown room (2). It tries to perform its task without success and finally get frustrated (3). The supervisor shows how to escape the room, afterward the system is able to escape autonomously.

The second lap corresponds to an intense metalearning stage since the predictor starts to learn and each place is new for the system (see Figure 13A). Distress level (Dl) shows peaks for each place. The Dl decreases while ending the loop, because the starting point is already predicted.

After this training stage, we let the robot performing its task for a while, correcting its trajectory only when needed (see Figure 13B). In this normal situation the distress level stays low (below 0.5) except for one area where it shows peaks. Such an area corresponds to a place where the robot navigates close to the window and is disturbed by the sunset at the time of the experiment. It means that the robot is less confident in its place/action strategy in this area. However it is not sufficient to frustrate the robot and the behavior stays coherent.

Later, the robot performs its task when the teacher suddenly deviates it into a small and unknown room (see Figure 13C). As the robot becomes lost, the distress level increases and finally reaches the frustration threshold. The robot stops and call for help. The teacher then assists the robot in escaping the room. The system learns that new state, gets out of the room and goes back to its stable attractor. The area where the robot were unconfident is now totally predicted since the sun is down and does not disturb it anymore.

## 4. Discussion

In this paper, we have addressed two different roles of a self-assessment mechanism for long range and complex robotic navigation. We presented its regulatory role in managing behaviors according to the situation, and its social role in communicating frustration to avoid deadlocks and improve learning.

First of all, we briefly presented our previous model of place cells that allows robots to perform simple and robust sensory-motor behaviors in small size environments. We highlighted the need to find solutions to overcome some issues we met while trying to navigate in more complex ones. We underlined situations where the number of available landmarks in the panorama is very low and the visual system deals with noisy information. We proposed to overcome these issues by taking into account other strategies. We extended our architecture by adding a robust and biologically plausible road following strategy that allows the robot to naturally converge to visible roads. Such a strategy allows to follow potential vanishing points instead of learning place/action associations, in situations where it is neither necessary, nor efficient to do so.

These behaviors defined the robot's skills for facing the situations encountered in the environment. However, these behaviors are in competition. The robot needs a second-order controller to manage them. Such a metacontroller needs a mechanism that evaluates behaviors. We argue that for evaluating its behavior, the system requires to monitor novelty in its predictions. Monitoring novelty or abnormality in the behavior is thus identified as a key feature for a second-order controller to manage robot's strategies. Following this statement, we proposed a model for self-assessment based on novelty detections in a dynamical point of view. In this view, the system must, at first, (1) learn to predict its sensations from its past perception in a training situation, next (2) monitor novelty and respond accordingly. We defined perception as an internal model of the sensory-motor interactions the system has with the external world. This model of perception provides a generic grounding to perform predictions on agent's sensation. The model could be adapted to any sensation/action loop and thus for a reasonable computational cost if one considers that, most of the time, sensation and action are correlated (except pathological cases). However, since the dimension of the “Perception” tensor might be large, it is important to define abstract input vectors (e.g. few landmark neurons instead of raw visual data) to avoid combinatorial explosion.

Even if we choose in this paper to stay at a theoretical level, the analogy with the computation that could be performed in the hippocampal system are strong enough to provide solutions to avoid scaling issues. In future works, we plan to replace the complete Per matrix by a sparse matrix where the encountered products could be learned by specific units (see our work on parahippocampal and perirhinal merging in prph (Giovannangeli et al., 2006)). To go one step further, the number of states could be also reduced if we avoid “grand mother” cells solution (both for landmarks and place cells) and replace them by a sparse coding allowing to use combinatorial aspects at our advantage. Another solution could be to represent the sensory activity by a compressed code having a low probability of ambiguity (something similar to a hach code or a random *M* to *N* projection with *M* >> *N* for instance).

It should also be noted that the model by itself could not learn different timing or periodic phenomena since the recurrent weight α has to be set empirically for each sensory-motor behavior. But the problem should be solved if we consider a set of novelty detectors with different time constants.

Then, we proposed to estimate the different moments about the mean by an online least-mean-square algorithm. One can note that least-mean-square requires independent and identically distributed (iid) random examples to ensure its convergence. However, even if such iid examples are not available in our experiments, the iid constraint is negligible here. Indeed, thanks to the large dimension of the perception matrix, (1) the problem is linear (except pathological cases) and (2) the system does not care about any complex unlearning processes nor does it need a high precision on its output.

Novelty is then measured based on the deviation of the monitored perception from the expected one. It is defined as the prediction error at a *n*'th level. Here, we defined novelty as the third order moment about the mean for empirical reasons. Actually, one can choose an arbitrary order to define novelty, depending on the desired accuracy. For example, modeling novelty by a first order error (simple difference between raw sensation and its average) results in a rather poor detection but decreases the time needed by the predictor to learn to predict the task. With such a poor system, periodical or sporadic events generates novelty as they differ from the average. Thus, they cannot be considered as normal by the system since variance is not taken into account. Conversely, defining novelty as a high order error results in a finer detection. However, in this case, the predictor needs a lot of time to completely predict a normal sensory-motor situation. Because of the online and memory-less constraints of our model, the estimation of a particular moment requires to wait for the estimation of each previous order. It raises few questions: Do animals predict in the same way? And do they have some estimation latency which increases by the level of precision?

Results showed that our novelty detection model presents good generalization capabilities since the same architecture can work at least for two different sensory-motor strategies.

Finally we showed that only considering the absolute novelty level was not sufficient to take correct decisions and regulate behaviors. For example, short perturbations might not affect so much the behavior. Most of the time, the robot stays in its attraction basin and performs the task correctly, even if unexpected events appear. The reason is that novelty does not refer by itself to a positive or negative reward. We showed that it is more interesting to consider the evolution of such a novelty activity rather than its absolute level. Monitoring the novelty tendency, by integrating its activity over time, provides a solution for the system to judge its own behavior. If the prediction error stagnates at a high level or if it increases whatever the robot tries, then the behavior should be considered as inefficient. And if this inefficiency is lasting this means the agent is caught in a deadlock.

Following these assumptions, we propose an emotional metacontroller (modeling frustration) that monitors prediction progress to modulate both strategies and adapt the behavior to the situation. We made several experiments that highlight the need for such a metacontroller to switch between strategies.

Moreover, we underline the role of emotions in communication by adding a simple distress call procedure triggered by the robot's frustration. This procedure allows the robot to communicate its inability to achieve the task by calling for help if no relevant strategies are found (if switching strategy does not increase any progress at all). Even if this procedure uses an ad-hoc distress call mechanism, it is triggered by a meaningful signal that point out situations where a refinement is possible. However, our emotional controller is not sufficient by itself to reach a full autonomy. Unlike intrinsically motivated systems such as (Schmidhuber, 1991; Barto et al., 2004; Oudeyer et al., 2007; Schembri et al., 2007; Baranes, 2011; Santucci et al., 2012), our system still requires a teacher to learn from demonstration but not in a prescriptive way. In this paper, frustration is presented as a useful intrinsic motivation for the agent to gradually develop its autonomy in an open-ended but supervised manner. Future works will focus on how to make use of this internal signal to improve learning in a fully autonomous way (without the need for human supervision).

Yet, our model has 3 main drawbacks that we should solve in a near future:

- The recurrent weight α, that defines the short-term-memory of the agent's perception, has to be different from one strategy to an other. Indeed, it highly depends on the own dynamic of the strategy involved.
- The size of the sensation vector *y* has a direct impact on the prediction dynamics. Indeed, the impact of a sensation neuron *y*_*i*_ on the novelty level is divided by the number of neurons in *y*.
- Yet, the learning stage is still separated from the use stage. This leads to the first role of emotions that could allow the agent to directly regulate its learning. The system should then decide whether to learn a situation as normal or abnormal.

Current works focus on testing the model performance on long range outside experiments (navigating several kilometers) with a real outdoor robot. We also focus on how a simple feedback loop can help the system to disambiguate its perception in an active way. Because of the ambiguity of perception, our system sometimes needs changes in its sensation to be able to correctly measure the quality of a given strategy. Thus, we study how to use the prediction error as a feedback signal that modulates actions accordingly. A high prediction error will trigger a high noise on robot's actions, inducing changes in sensation. Such changes will decrease the prediction error only if sensory-motor contingencies become predictable, and will increase it if not. Behavioral alteration is directly proportional to the prediction error. We wish this homeostatic mechanism will allow the system to regulate itself, updating its knowledge by actively altering its behavior in order to check whether its expectation is true.

### Conflict of interest statement

The authors declare that the research was conducted in the absence of any commercial or financial relationships that could be construed as a potential conflict of interest.

## References

[B1] Amari S. (1977). Dynamics of pattern formation in lateral-inhibition type neural fields. Biol. Cybern. 27, 77–87 10.1007/BF00337259 911931

[B2] Andry P. Gaussier P. Nadel J. (2002). From Visuo-Motor Development to Low-level Imitation, in Proceedings of the second workshop on Epigenetic Robotics, (Lund: Lund University Cognitive Studies), 94.

[B3] Andry P. Gaussier P. Nadel J. Hirsbrunner B. (2004). Learning invariant sensory-motor behaviors: a developmental approach of imitation mechanisms. Adapt. Behav. 12, 117–140 10.1177/105971230401200203

[B4] Banquet J.-P. Gaussier P. Quoy M. Revel A. Burnod Y. (2005). A hierarchy of as- sociations in hippocampo-cortical systems: Cognitive maps and navigation strategies. Neural Comput. 17, 1339–1384 10.1162/0899766053630369 15901401

[B5] Baranes A. (2011). Motivations Intrinseques et Contraintes Maturationnelles pour l'Apprentissage Sensorimoteur. PhThesis, D, INRIA - Sud Ouest: Université de Bordeaux

[B6] Barto A. Singh S. Chentanez N. (2004). Intrinsically motivated learning of hierarchical collections of skills, in International Conference on Developmental Learning (ICDL), La Jolla

[B7] Bishop C. M. (1994). Novelty detection and neural network validation. IEEE Proc. Vis. Image Signal Process. 141, 217–222 10.1049/ip-vis:19941330

[B8] Braitenberg V. (1986). Vehicles: Experiments in Synthetic Psychology. Cambridge: MIT Press

[B9] Carpenter G. A. Grossberg S. (1988). The ART of adaptive pattern recognition by a self-organising neural network. IComputer EEE 21, 77–88

[B10] Caluwaerts K. Staffa M. N'Guyen S. Grand C. Dollé L. Favre-Félix A. (2012). A biologically inspired meta-control navigation system for the Psikharpax rat robot. Bioinspir. Biomim. 7:025009 10.1088/1748-3182/7/2/025009 22617382

[B11] Cuperlier N. Gaussier Ph. Laroque Ph. Quoy M. (2005). Goal and motor action selection using an hippocampal and prefrontal model, in Model. Nat. Action Select. (Edinburgh: AISB Press), 100–106

[B12] Cuperlier N. Quoy M. Gaussier Ph. (2007). Neurobiologically inspired mobile robot navigation and planning. Front. Neurorobot. 1:3 10.3389/neuro.12.003.2007 18958274 PMC2533588

[B13] Damasio A. (2003). Looking for Spinoza: Joy, Sorrow and the Feeling Brain. San Diego, CA: Harcourt Inc

[B14] Devroye L. Wise G. L. (1980). Detection of abnormal behavior via nonparametric estimation of the support. Appl. Am. J. Math. 38, 480–488 10.1137/0138038

[B15] Dollé L. (2011). Contribution d'un Modele Computationnel de Sélection de Stratégies de Navigation aux Hypotheses Relatives á l'apprentissage Spatial, Paris: PhThesis, D, UPMC-Sorbonne univeristés

[B16] Fong T. W. Nourbakhsh I. Dautenhahn K. (2003). A survey of socially interactive robots. Robot. Auton. Syst. 42, 143–166 10.1016/S0921-8890(02)00372-X

[B17] Gaussier P. Zrehen S. (1995). Perac: a neural architecture to control artificial animals. Robot. Autonom. Syst. 16, 291–320 10.1016/0921-8890(95)00052-6

[B18] Gaussier P. Revel A. Banquet J.-P. Babeau V. (2002). From view cells and place cells to cognitive map learning: processing stages of the hippocampal system. Biol. Cybern. 86, 15–28 10.1007/s004220100269 11918209

[B19] Gaussier P. Baccon J. C. Prepin K. Nadel J. Hafemeister L. (2004). Formalization of recognition, affordances and learning in isolated or interacting animats, in The Society for Adaptive Behavior SAB'04, (Los Angeles, CA: MIT Press), 57–66

[B20] Giovannangeli C. Gaussier P. Banquet J. P. (2006). Robustness of visual place cells in dynamic indoor and outdoor environment. Int. J. Adv. Robot. Syst. 3, 115–124 10.5772/5748

[B21] Gibson J. (1979). The Ecological Approach to Visual Perception. Boston, MA: Houghton Mifflin

[B22] Grandjean D. Peters C. (2011). Novelty processing and emotion: conceptual developments, empirical findings and virtual environments, in Emotion-Oriented Systems: The Humaine Handbook, eds Petta P. Pelachaud C. Cowie R. (London: Springer), 441–458

[B23] Griffiths P. E. (1997). What Emotions Really Are: The Problem of Psychological Categories. Chicago, IL: University of Chicago Press 10.7208/chicago/9780226308760.001.0001

[B24] Grossberg S. (1972a). A neural theory of punishment and avoidance. I. Qualitative theory. Math. Biosci. 15, 39–67 10.1016/0025-5564(72)90062-4

[B25] Grossberg S. (1972b). A neural theory of punishment and avoidance. II. Quantitative theory. Math. Biosci. 15, 253–285 10.1016/0025-5564(72)90038-7

[B26] Guillot-Gurnett A. K. N. Girard B. Cuzin V. Prescott T. J. (2002). From animals to animats 7, in Proceedings of the Seventh International Conference on Simulation of Adaptive Behavior. eds Hallam-Hayes J. Hallam G. B. Floreano D. Mayer J. A.(Cambridge: MIT Press).

[B27] Gumbel E. J. (1958). Statistics of Extremes, New York, NY: Columbia University Press

[B28] Hasselmo M. E. McClelland J. L. (1999). Neural Models of Memory, Vol. 9, (Boston: Elsevier), 0959-(4388) 10.1016/S0959-438880025-438880027 10322183

[B29] Hasson C. Gaussier P. Boucenna S. (2011). Emotions as a dynamical system: the interplay between the meta-control and communication function of émotions. J. Behav. Robot. 2, 111–125

[B30] Hirel J. Gaussier P. Quoy M. Banquet J. P. Save E. Poucet B. (2013). The hippocampo-cortical loop: spatio-temporal learning and goal-oriented planning in navigation. Neural Netw. 43, 8–21 10.1016/j.neunet.2013.01.023 23500496

[B31] Jauffret A. Grand C. Cuperlier N. Gaussier P. Tarroux P. (2013). How can a robot evaluate its own behaviour? A generic model for self-assessment, in International Joint Conference on Neural Networks (IJCNN) (Dallas, TX).

[B32] Kaski S. Kangas J. Kohonen T. (1981). Bibliography of self-organising map (SOM) papers: - (1997). Neural Comput. Surveys 1, 102–350

[B33] Kelley S. Brownell C. Campbell S. (2000). Mastery motivation and self-evaluative affect in toddlers: longitudinal relations with maternal behavior. Child Dev. 71, 1061–1071 10.1111/1467-8624.00209 11016566

[B34] Knight R. T. (1996). Contribution of human hippocampal region to novelty detection. Nature 383, 256–259 10.1038/383256a0 8805701

[B35] Kohonen T. Oja E. (1976). Fast adaptive formation of orthogonalizing filters and associative memory in recurrent networks of neuron-like elements. Biol. Cybern. 25, 85–95 10.1007/BF01259390 1244872

[B36] Laroque Ph. Gaussier N. Cuperlier N. Quoy M. Gaussier Ph. (2010). Cognitive map plasticity and imitation strategies to improve individual and social behaviors of autonomous agents. J. Behav. Robot. 1, 25–36 10.2478/s13230-010-0004-2

[B37] Lazarus R. (1991). Emotion and Adaptation. New York, NY: Oxford University Press

[B38] Levine D. S. Prueitt P. S. (1992). Simulations of conditioned perseveration and novelty preference from frontal lobe damage, in Neural Network Models of Conditioning and Action, Chapter 5, eds Commons M. L. Grossberg S. Staddon E. R. J. (Hillsdale, NJ: Lawrence Erlbaum Associates), 123–147

[B39] Lewis D. (2005). Bridging emotion theory and neurobiology through dynamic systems modeling. Behav. Brain Sci. 28, 169–194; discussion: 194–245. 10.1017/S0140525X0500004X 16201458

[B40] Lisman J. E. Otmakhova N. A. (2001). Storage, recall, and novelty detection of sequences by the hippocampus: elaborating on the SOCRATIC model to account for normal and aberrant effects of dopamine. Hippocampus 11, 551–568 10.1002/hipo.1071 11732708

[B41] Maillard M. Gapenne O. Hafemeister L. Gaussier P. (2005). Perception as a dynamical sensori-motor attraction basin (2005), in Advances in Artificial Life (8th European Conference, ECAL). Vol. 3630, (Canterbury), 37–46

[B42] Marsland S. (2002). Novelty detection in learning systems. Neural Comput. Surv. 3, 1–39

[B43] Mirolli M. Baldassarre G. (2013). Functions and mechanisms of intrinsic motivations: The knowledge vs. competence distinction, in Intrinsically Motivated Learning in Natural and Artificial Systems. eds Baldassarre G. Mirolli M. (Berlin: Springer-Verlag), 49–72

[B44] O'Keefe J. Nadel N. (1978). The Hippocampus as a Cognitive Map. Oxford: Clarendon Press

[B45] Oudeyer P.-Y. Kaplan F. Hafner V. (2007). Intrinsic motivation systems for autonomous mental development. IEEE Trans. Evol. Comput. 11, 265–286 10.1109/TEVC.2006.890271

[B46] Pavlov I. P. (1927). Conditioned Reflexes: An Investigation of the Physiological Activity of the Cerebral Cortex. Anrep G. V., Trans. ed, London: Oxford University Press 10.5214/ans.0972-7531.1017309 PMC4116985 25205891

[B47] Prescott T. J. Gurnett K. Redgrave P. (2001). A computational model of action selection in basal ganglia. i. a new functional anatomy. Biol. Cybern. 84, 410 11417052 10.1007/PL00007984

[B48] Quoy M. Moga S. Gaussier P. (2003). Dynamical neural networks for top-down robot control. IEEE Trans. Man Syst. Cybern. A 33, 523–532 10.1109/TSMCA.2003.809224

[B49] Richefeu J. Manzanera A. (2006). A new hybrid differential filter for motion detection. Comput. Vis. Graph. 32, 727–732

[B50] Roberts S. Tarassenko L. (1994). A probabilistic resource allocating network for novelty detection. Neural Comput. 6, 270–284 10.1162/neco.1994.6.2.270

[B51] Santucci V. Baldassarre G. Mirolli M. (2012). Intrinsic motivation mechanisms for competence acquisition, in Development and learning and epigenetic robotics (ICDL), IEEE Int. Conf. (San Diego, CA), 1–6 10.1109/DevLrn.2012.6400835

[B52] Scherer K. R. (1984). On the nature and function of emotion. A componentprocess approach, in Approaches to Emotion, eds Scherer K. R. Ekman P. (Hillsdale: Erlbaum), 293–317

[B53] Schembri M. Miroll M. Baldassarre G. (2007). Evolving childhood's length and learning parameters in an intrinsically motivated reinforcement learning robot, in Proceedings of the Seventh International Conference on Epigenetic Robotics (EpiRob). (Lund: Lund University Cognitive Studies), 141–148

[B54] Schmidhuber J. (1991). Curious model-building control system, in Proceedings of International Joint Conference on Neural Networks Vol. 2, (Singapore: IEEE), 1458–1463

[B55] Schoner G. Dose M. Engels C. (1995). Dynamics of behavior: theory and applications for autonomous robot architectures. Robot. Autonom. Syst. 16, 213–245

[B56] Sidak Z. Pranab Sen K. Hajek J. (1967). Theory of Rank Tests. 2nd edition (San Diego, CA: Academic Press), 435.

[B57] Stipek D. Recchia S. McClintic S. (1992). Self-evaluation in young children. Monogr. Soc. Res. Child. Dev. 57, 1–98 10.2307/1166190 1560797

[B58] Taylor S. E. Neter E. Wayment H. A. (1995). Self-evaluation processes. Pers. Soc. Psychol. Bull. 21, 1278–1287 10.1177/01461672952112005

[B59] Tronick Z. (1989). Emotions and emotional communication in infants. Am. Psychol. 44, 112–119 10.1037/0003-066X.44.2.112 2653124

[B60] Widrow B. Hoff M. E. Jr. (1960). Adaptive Switching Circuits. IWES Convention Record, RE, CON 4, (Stanford, CA), 96–104

[B61] Zillich M. Prankl J. Morwald T. Vincze M. (2011). Knowing your limits - self-evaluation and prediction in object recognition, Intelligent Robots and Systems (IROS), in IEEE/RSJ International Conference, (San Francisco, CA), 813–820 10.1109/IROS.2011.6094856.

